# Health systems influence on the pathways of care for lung cancer in low- and middle-income countries: a scoping review

**DOI:** 10.1186/s12992-020-00553-8

**Published:** 2020-03-18

**Authors:** Ugochinyere I. Nwagbara, Themba G. Ginindza, Khumbulani W. Hlongwana

**Affiliations:** Discipline of Public Health Medicine, School of Nursing and Public Health, University of KwaZulu-Natal, Durban, 4041 South Africa

**Keywords:** Lung cancer, Screening, Diagnosis, Health care system, Low-and middle-income countries

## Abstract

**Background:**

Globally, lung cancer is the most common cancer and cause of cancer-related deaths, responsible for nearly one in five deaths. Many health systems in low- and middle-income countries, including sub-Saharan Africa have weak organizational structure, which results in delayed lead time for lung cancer patient care continuum from diagnosis to palliative care.

**Aim:**

To map evidence on the health systems issues impacting on the delays in timely lung cancer care continuum from diagnosis to palliative care in LMICs, including sub-Saharan Africa.

**Methods:**

A scoping review was performed following the method of Arksey and O’Malley. Systematic searches were performed using EBSCOhost platform, a keyword search from the following electronic databases were conducted: PubMed/MEDLINE, Google Scholar, Science Direct, World Health Organization (WHO) library, and grey literature. The screening was guided by the inclusion and exclusion criteria. The quality of the included studies was determined by Mixed Method Appraisal Tool (MMAT).

**Results:**

A total of 2886 articles were screened, and 236 met the eligibility criteria for this scoping review study. Furthermore, 155 articles were also excluded following abstract screening. Eighty-one articles were selected for full-article screening by two researchers with 10 being selected for independent detailed data extraction for synthesis. These studies were also subjected to methodological quality assessment. All included studies were conducted in LMICs mostly Asia, the Middle East, and Latin America and published between January 2008 and June 2018. The ten included studies described at least one interval in lung cancer care.

**Conclusions:**

Reducing wait time across this care continuum is needed to improve easy access to healthcare, quality care, survival and patient outcomes, as many patients still face longer wait times for diagnosis and treatment of lung cancer than recommended in several healthcare settings. A multidisciplinary team approach will help to reduce wait time and ensure that all patients receive appropriate care. Interventions are needed to address delays in lung cancer care in LMICs. Health-care providers at all levels of care should be educated and equipped with skills to identify lung cancer symptoms and perform or refer for appropriate diagnostic tests.

## Background

Cancer is the second leading cause of death after cardiovascular diseases worldwide and an emerging public health problem in sub-Saharan Africa (SSA) [1]. Globally, more than 20 million new cancer cases are projected for 2025 compared to an estimated 14.1 million and 17.5 million new cancer cases in 2012 and 2015 respectively [1–4]. The newly adopted Sustainable Development Goals (SDGs) focuses on the target for universal health coverage (UHC) to achieve the development of a strong health system [5–7]. The growing burden of non-communicable diseases (NCDs), including cancer on low- and middle-income countries (LMICs) has become a threat to the already weakened health system. The health workers and health system are least prepared to manage this burden which continues to grow, exerting tremendous physical, emotional and financial strain on individuals, families, communities and health system in general [8].

The WHO defined the health system as consisting of all organizations, people and actions whose primary purpose is to promote, restore and maintain health [9]. Cancer care in LMICs will require a strong health system that spans prevention, early diagnosis, surgery and radiation capabilities, drug delivery, patient support, and palliative care [10].

Lung cancer is the most common cancer and cause of cancer-related deaths across the globe, responsible for nearly one in five deaths [11], and the most commonly diagnosed cancer worldwide (1.8 million) [12]. About 715,000 new cancer cases and 542,000 cancer deaths occurred in Africa and these numbers are expected to double in the next 20 years [13]. The anticipated increases are associated with the aging and growth of the population as well as the changes in lifestyle factors associated with urbanization and economic development of risk factors such as smoking, obesity, physical inactivity and dietary patterns [4, 13].

Late stage at diagnosis for lung cancer results in delays that may adversely affect survival so rapid diagnosis and treatment are important [14, 15]. Improving early diagnosis capacity is a significant strategy to cancer control and strengthening of the health systems [8]. Other health system factors that potentially impact on delay in timely lung cancer diagnosis include: long-wait times for initial assessment and waits between procedures, schedule inflexibility and poorly communicated processes, which often result in a missed appointment [16]. In LMICs, including SSA, most people are diagnosed with advanced lung cancer due to poor access to care, lack of awareness, inadequate health-care infrastructures, and poor referrals to diagnosis and palliative care [8, 17]. Furthermore, cancer medications are hardly provided at reduced rates by the governments, thereby making treatments unaffordable for the poor [17], and this also tends to involve a greater economic burden for families [18]. The SDGs target for UHC emphasizes the importance of all people and communities in having access to quality health services without risking financial hardship [6, 7]. Health system strengthening is a means to progress towards UHC, including financial risk protection, access to quality essential health care services, and access to safe, effective, quality, and affordable essential medicines and vaccines for all [6, 7].

While prevention, screening, and palliative care efforts are an important strategy to reduce the global cancer burden, increased investment in health systems and access to medicines policy cannot be ignored [19]. Beyond medicines alone, effective health systems with highly trained medical professionals are critical to improving access to treatment [19–21]. Concentrating on increasing the capacity of health systems in LMICs which includes comprehensively training medical personnel can improve health outcomes [19].

Intervals between suspicion, diagnosis, and treatment of lung cancer vary widely among patients [22]. By providing timely care at all steps of the lung cancer care continuum, providers may be able to limit disease progression before treatment, increase patient satisfaction and possibly improve clinical outcomes [23].

As a result of late presentation to health facilities and little access to appropriate diagnostic technology, approximately 80% of the cases are diagnosed when they are already in terminal stages [24]. Timely diagnosis and treatment of lung cancer is critical because delays can lead to missed opportunities for both curative and life-prolonging therapies [25]. However, there is paucity of research evidence regarding health systems issues contributing to the delay in timely lung cancer care continuum from diagnosis to palliative care in sub-Saharan Africa. Implementing cost-effective cancer interventions across the care continuum can strengthen the health system [26]. The results of this study are anticipated to map evidence on the health systems issues impacting on the delays in timely lung cancer care continuum from diagnosis to palliative care in LMICs, including SSA, so that appropriately targeted interventions can be identified.

## Methods

This study was conducted through a scoping review. This approach is particularly appropriate when the main sources and types of available evidence are complex or have not been reviewed comprehensively before [27]. This review included a quality assessment as recommended by Levac et al. [28] and was guided by the 5-step methodological framework outlined by Arksey and O’Malley [29]. These steps consist of: [1] identifying the research question [2]; identifying the relevant studies [3]; study selection and eligibility [4]; charting the data, and [5] collating, summarizing and reporting the results.

### Identifying the research question

Our research question was “what is known from existing literature on the health systems issues impacting on the delays in timely lung cancer care continuum from diagnosis to palliative care in low-and middle-income countries?”

### Identifying relevant studies

To identify relevant studies, we performed a scoping review including all study designs published in peer-reviewed journals as well as in grey literature addressing the research question. The search was performed using EBSCOhost platform, a keyword search from the following electronic databases was conducted: PubMed/MEDLINE, Google Scholar, Science Direct, World Health Organization (WHO) library, and grey literature. Studies were identified by searching literatures published in English language as it is the commonly used language for communication in most SSA countries. We restricted the search to include studies published from January 2008 to June 2018 because initial searches of the literature showed that most relevant studies were conducted after 2008. Additionally, a 10-year literature search is more likely to yield a comprehensive account of previous and current research in the area. Articles were also searched through the ‘Cited by’ search as well as citations included in the reference lists of included articles. The search terms included Lung cancer, Diagnosis and Health care system. Boolean terms (AND, OR) were used to separate the keywords during the search. Medical Subject Headings (Mesh) terms were also included in the search. We hand searched eligible studies from the list of references of included studies. The search strategy is included in Additional file 1.

### Study selection and eligibility

Following title screening from the above-mentioned databases, articles with relevant study titles for this research were uploaded on the Endnote X7 software. Search results from different electronic databases were combined in a single EndNote library. Studies which did not address the research question and the duplicates of the same records were then excluded. Abstract and full articles were screened from the included studies by two independent reviewers (UIN and MO). An abstract screening form with questions was developed based on the review eligibility criteria. Discrepancies between reviewers at abstract and full article stages was resolved by involving a third screener. The relevant studies were identified with guidance from the inclusion and exclusion criteria which was formulated according to the research questions.

### Inclusion criteria

All studies included met the following inclusion criteria:

- Articles published in English.
- Published from January 2008 to June 2018.
- All study designs with relevant interventions.
- Studies focusing on lung cancer diagnosis to palliative care in adults.
- Research focusing on Health systems influence on the pathways of lung cancer in Low and Middle-Income Countries (LMICs) and whose conclusions and discussions demonstrate transferable and or generalizable findings to African settings.

### Exclusion criteria

Studies with the following characteristics were excluded:

- Articles published in other languages other than English.
- Studies published before January 2008.
- Articles focusing on Health systems influence on the pathways of lung cancer in High income countries.
- Studies focusing on lung cancer diagnosis to palliative care in children.

### Charting the data

Data on the study setting and the key findings described in each article were recorded and organized into different themes using NVivo 10. Information from the selected studies was sorted and organized into the following categories: author and year, country of origin, study aim, study population, study design, study setting, and most relevant findings.

### Collating, summarising and reporting the results

In the process of collating and summarizing the findings, the extracted evidence was repeatedly reviewed. Results were summarized to present an overview of the current evidence on health systems issues impacting on the delays in timely lung cancer care continuum from diagnosis to palliative care. We performed a thematic content analysis of the themes to identify further contextual factors (e.g. misdiagnosis for lung cancer, delays in timely diagnosis of lung cancer and referrals, Waiting time intervals, high cost and inaccessibility of diagnostic facilities etc).

### Quality assessment

Mixed Method Quality Appraisal Tool (MMAT) Version 2011 [30], was used for quality assessment of included studies for the purpose of evaluating the risk of bias. Two reviewers (UIN and MO) independently assessed the quality of evidence of the studies included. The studies were assessed in the following domains: the appropriateness of aim of the study, adequacy and methodology, study design, data collection, study selection, data analysis, presentation of findings, author’s discussions and conclusions. An overall quality percentage score for each of the included studies was calculated and scores interpreted as low quality (<50%), average quality (51–75%), and high quality (76–100%).

## Results

### Screening results

After the title screening and deletion of duplicates, this scoping review found 236 eligible studies from a total of 2886 articles. A total of 155 articles were also excluded following abstract screening. Eighty-one (81) articles were selected for full-article screening by two researchers with 10 being selected for independent t detailed data extraction for this synthesis. These studies were also subjected to methodological quality assessment. Cohen’s kappa coefficient (κ) statistic using Stata 13.0SE (Stata corp. College station, Texas, USA) was used to measure inter-rater agreement between reviewers [31], and the result shows that there was 80.00% agreement versus 82.00% expected by chance which constitutes a considerably poor agreement between screeners (Kappa statistic = − 0. 11 and *p*-value < 0.05). However, the McNemar’s chi-square statistic suggests that there is not a statistically significant difference in the proportions of yes/no answers by reviewer with *p*-value < 0.05. The degree of agreement calculation is included in Additional file 2.

**Fig. 1:**
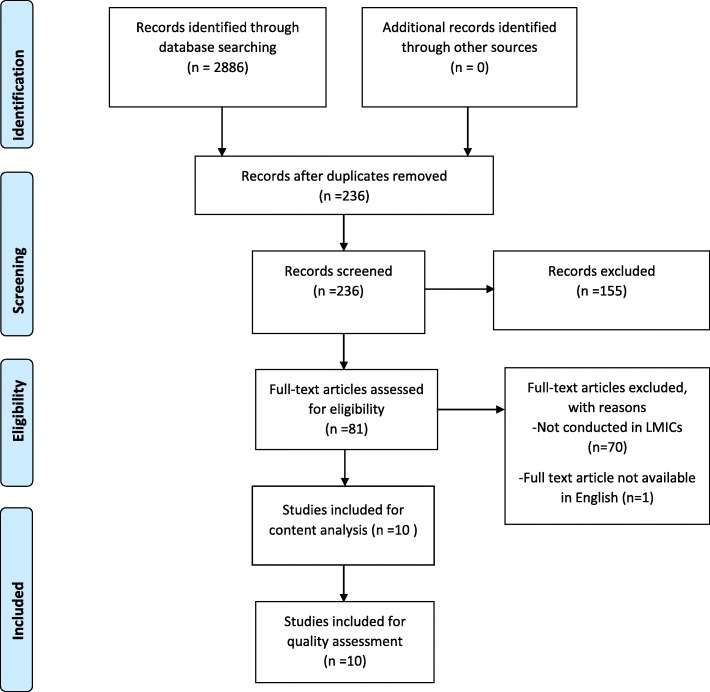
PRISMA record screening flow-chart. [Source: Adapted from Moher et al. [32]]

The Preferred Report Items for Systematic and Meta-Analysis (PRISMA) flow chart for the selection and screening of studies done in this research is shown in Fig. 1.

### Characteristics of included studies

All included studies were conducted in LMICs and published between January 2008 and June 2018. The total sample size from primary studies was 3133 participants. An average of 2233 were predominately male from nine studies and 803 females [33–41], one study [42] did not specify on the sex of the participants. About smoking status, six studies showed significant male predominance [33–35, 38, 40, 41]. Of the ten included studies, 4 were retrospective studies [33, 34, 36, 41]; 3 were prospective studies [38, 40, 42]; 2 were descriptive studies [37, 39] and 1 was cross-sectional study [35]. All the ten studies described at least one interval in lung cancer care. Three studies reported that most patients visited two or more GPs before a confirmation of their diagnosis [37, 40, 42]. Eight studies showed median time from symptom onset to diagnosis [33–35, 37–40, 42]. Seven studies showed reasons for misdiagnosis and misinterpretations of findings among the GPs [34, 35, 37–40, 42]. Characteristics of the included studies are presented in Table 1.

**Table 1:** Characteristics of included studies

Author and year	Study setting (Country)	Aim	Study design	Population (n)	Mean/Age range of participants	Percentage of males	Percentage of females	Health systems factors that impact on delay in timely lung cancer diagnosis	Relevant findings
Abrao, F. C. and 2017 [33]	Brazil	To evaluate the access of patients with lung cancer in a densely populated area of São Paulo to the Brazilian Public Health System, focusing on the time spent from symptom onset or initial diagnosis until the beginning of treatment.	Retrospective study	509	62.50 years	57.8%	42.2%	Long waiting times and health system’s incapacity to absorb all patients.	Diagnosis is faster when the patient can make an appointment with a specialist
Chandra, Subhash and 2009 [34]	India	To determine the average time period required at various steps for diagnosing lung cancer from the onset of symptoms at a tertiary referral centre in Northern India	Retrospective study	165	53 years and older	84.2%	15.8%	High cost and inaccessibility of diagnostic investigations such as CT scan, chemotherapy, broncoscopy and long waiting periods	Inappropriate treatment with ATT significantly prolongs delay
Chatterjee, Surajit and 2016 [35]	India	To summarize the contributing factors in delaying diagnosis of lung cancer for our better knowledge.	Observational cross-sectional	50	55.50 years	72.0%	28.0%	High cost and limited availability of CT thorax and long waiting times	Patients were not advised on CT thorax at appropriate time and instead received ATT causing referral delay
Fernandez de la Vega, J. F. and 2015 [35]	Cuba	To Assess diagnostic delay of lung cancer in patients at the Joaquín Albarrán Clinical-Surgical Teaching Hospital, Havana, Cuba, from 2007 to 2010.	Retrospective study	54	51–60 years	74.1%	25.9%	Lack of diagnostic tools for imaging, endoscopy and pathology, lack of suitable lung cancer protocol, poor organization and management of health services.	Patients who went directly to hospital did not benefit from shorter delay in diagnosis
Hsieh, Vivian Chia-Rong and 2012 [25]	Taiwan	To understand the delay in the diagnosis of lung cancer under the healthcare system in Taiwan, and to identify the factors associated with it.	Descriptive analysis	840	62.68 years	55.9%	44.1%	Misinterpretation of a chest CT scan, high cost of CT scan and delays due to multiple medical visits.	Delays increase as number of doctors or visits increase
Ramachandran, Krishnappriya and 2016 [40]	India	To assess physician related delays in the diagnosis of lung cancer and the treatments given before presenting to our center.	Prospective study	96	Not specified	Not specified	Not specified	Lung cancer was misdiagnosed as TB and treated with ATT, inadequacy of medical services, delay in referrals and in the performance of subsidiary tests.	Many physicians have a low index of suspicion to diagnose lung malignancy and most commonly misdiagnose it as tuberculosis
Sulu, E. and 2011 [36]	Turkey	To investigate patterns of delays among patients with non-small-cell lung cancer and to identify reasons for the delays.	Prospective study	101	60.6 years	90.1%	9.9%	Low index of suspicion for lung cancer, organizational problems, long waiting lists for radiotherapy and surgery, low performance of diagnostic methods and limited	Prehospital delays are largely dependent on the level of patient education and complex socioeconomic factors.
Valdes, S. and 2010 [37]	Cuba	To identify the length of diagnostic delay in a group of patients diagnosed with non-small cell lung cancer by determining the time elapsed from onset of symptoms to confirmation of diagnosis.	Descriptive observational study	96	32–88 years	69.0%	31.0%	Delays in obtaining results of CT tests, low index of suspicion for lung cancer and unavailability of diagnostic tools.	The length of health system-attributed diagnostic delay was prolonged and in need of improvement.
Yurdakul, Ahmet Selim and 2015 [38]	Turkey	To investigate patient and doctor delays in the diagnosis and treatment of NSCLC and the factors affecting these delays.	Prospective study	1016	61.5 years	91.0%	8.9%	Physician’s opinion of another diagnosis, delays in pathologic/radiologic/bronchoscopic examination, patient refusal to undergo procedure, a large volume of patients/lack of bed and long waiting time for hospitalisation.	Diagnostic and treatment delays were longer in early-stage cases.
Zivkovic, D. and 2014 [39]	Montenegro	To investigate whether waiting times and delays in diagnosis and treatment of patients with lung carcinoma have any bearing on prognosis and survival.	Retrospective study	206	66 years	83.0%	17.0%	Specialist services delay.	No significant difference in survival between patients with stage I and stage II NSCLC according to delay from the onset of first symptoms.

### Quality of evidence from included primary studies

All of the 10 included primary studies went through quality assessment using the Mixed Methods Appraisal Tool (MMAT) – Version 2011 [30]. The studies were assessed based on all the categorized domains. All the ten included studies had high quality percentage of 76–100% ( [33–42]. None of the ten included studies for quality assessment scored low quality (<50%) percentage. The overall evidence was considered to have minimal risk of bias. Quality assessment of included studies are presented in Additional file 3: Table 1.

### Health systems factors impacting on the delays in timely lung cancer diagnosis

#### Patient delay

The most common reason for patient’s delay was ignoring the symptoms by the patients [38]. A study conducted by Abrao et al. in Brazil reported that most patients (68.3%) came to the hospital when the disease was already at an advanced stage III and IV [35] and another study showed that at the time of diagnosis, (90.2%) of the Non-small cell lung cancer (NSCLC) patients had stage IIIB or IV disease [36]. Most of these patients began receiving treatment at about 1.5 months post diagnosis [35]. A decrease in survival was observed in patients who started treatment when the disease was at advanced stages despite being prioritized to start treatment sooner than those at lower stages [35]. This is contrary to a study conducted in Montenegro, which showed that prognoses was worse in patients with shorter delay and that patients with limited disease had longer delay until they received cancer specific treatment than those with advanced disease [42]. Most patients failed to seek the services of pulmonary specialists directly or through referral either due to a shortage of pulmonary specialists or due to other reasons [42].

### Physician delay

Seven studies reported delays due to misdiagnosis of lung cancer as tuberculosis and misinterpretation of chest CT scan by the doctors [34, 35, 37–40, 42]. Two studies concluded that a low index of suspicion for lung cancer was the most common cause for referral delay [38, 42]. Three studies reported that delay in diagnosis was significantly higher in patients who had received antitubercular treatment (ATT) after lung cancer was misdiagnosed as tuberculosis [34, 35, 42]. A study performed in Turkey, reported that the reasons for doctor delays was insufficient knowledge of lung cancer by the physicians who were involved in the monitoring of patients with lung cancer, incorrect assessment of lung radiographs, performing unnecessary diagnostic procedures and insufficiencies of medical services and laboratories [40].

Other physician-related delays emanated from the multiple physician consultations patients were subjected to, before proper referrals could be made. Four studies demonstrated that patients often made multiple visits to their primary care physician and had twice as many GP consultations before a confirmation of their diagnosis. As the number of hospital visits increased, the delay in diagnosis also increased [37, 38, 40, 42].

### Health system delay

Patients spent a lot of time waiting during each interval period due to the health system’s incapacity to absorb all patients [33]. A study conducted in Brazil [33], showed that the median time from symptom onset to diagnosis was 3 months (90 days), another study conducted at All India Institute of Medical Sciences [34] showed that the delay between the onset of symptoms to confirmed diagnosis was 143 days. The duration of delay from onset to diagnosis was shorter in most studies except in a study conducted in India [42] which showed the median time from the onset of symptoms to diagnosis to be around 6 months.

A study conducted in Turkey [38] showed that the median application interval which is the interval between onset of symptom to the first doctor visit was 25 days whereas another study conducted in India [35] presented a longer median application interval to be 94 days.

The British Thoracic Society (BTS) recommends that all patients should be seen for an initial evaluation by a pulmonary physician within 1 week of referral from their primary care physician and, diagnostic testing should be performed within 2 weeks of the decision [43]. In the Canadian recommendations, a maximum of 4 weeks lapse between the first visit to a general practitioner and diagnosis is considered acceptable, and the waiting time for surgery should not exceed 2 weeks [44].

Seven studies reported that the high cost and inaccessibility of diagnostic investigations such as CT scan, bronchoscopy, chemotherapy, chest radiography may contribute to their inadequate utilization and in turn result to delays in the initiation of treatment even after the diagnosis has been established [34–37, 39, 40, 42]. A study reported that the reason for referral delay was due to the unavailability of a particular diagnostic tool and this resulted to delays in obtaining results of examinations not performed on site, such as computerized axial tomography, or to patients’ postponement or refusal of invasive procedures, such as bronchoscopy and transparietal fine-needle aspiration biopsy [39].

## Discussion

This scoping review mapped available literature on the health systems issues impacting on the delays in timely lung cancer care continuum from diagnosis to palliative care, which is of primary importance to inform timely lung cancer diagnosis in LMICs, including SSA.

This study was intended to focus on the SSA countries. However, due to the paucity of literature on the health systems issues impacting on the delays in timely lung cancer care continuum from diagnosis to palliative care in SSA, we extended our study setting to include studies from LMICs and whose conclusions and discussions demonstrate transferable and or generalizable findings to African settings. Despite the differences in social and ethnic setting within the LMICs, our findings suggest that the LMICs largely have common health systems issues impacting on the delays in timely lung cancer diagnosis to palliative care.

In this review, we identified 10 articles published between 2009 and 2017 that recognized the health systems issues impacting on the delays in timely lung cancer care continuum from diagnosis to palliative care in LMICs mostly Asia, the Middle East, and Latin America [33–42]. Patients in LMICs do not have access to effective community education, preventive services, screening and early detection, surgical or adequate primary health care, and thus, tend to come to health centers and hospitals with late stage cancer diagnoses that are incredibly difficult to treat [19, 45]. All the reviewed studies echoed the urgent need to educate the physicians and the public about symptoms of lung cancer and promote early diagnosis for more effective and less expensive treatment. This study revealed that Pulmonology specialist appeared to be the most likely to suspect lung cancer compared to general practitioners [42]. An average of 2233 was predominately male from nine studies and 803 females [33–41], this predominance of men from our results are consistent with literature that lung cancer incidence is still higher in men than women [46].

Studies included in this review noted some health system factors responsible for the delays in timely lung cancer diagnosis, such as misdiagnosis for lung cancer and delays due to referrals by the physicians, the waiting time intervals, high cost and inaccessibility of diagnostic facilities [33–42]. Poor knowledge and ignorance of the lung cancer symptom, inaccessibility to health services, lack of awareness and failure to recognise symptoms, negative beliefs about cancer outcomes and fear of consultation are some of the factors that lead to patients delay in seeking medical attention [15, 39, 47, 48]. Some studies stated that poor organization and management of health services play an important role in diagnostic delay and that the health system delays are influenced by health worker expertise in symptom recognition, availability and organization of facilities and resources [36, 38]. There is need for policy makers and GPs to strengthen the healthcare systems by ensuring that they integrate and scale up national lung cancer prevention and control as part of the national responses to non-communicable diseases which is in line with the 2030 Agenda for Sustainable Development.

The findings of this study, which is consistent with other studies conducted in LMICs, showed evidence that many patients across different facilities in LMICs are facing longer than recommended wait-times for lung cancer diagnosis and care. Valdés et al. found the health system to be the greater contributor (mean delay 61.6 days) of delay to timely lung cancer diagnosis [39]. British Thoracic Society (BTS) recommends that all patients should receive treatment in 31 days or less once diagnosed [43]. In LMICs, the health systems have a weak organisational structure, leading to uncoordinated activities at all levels of care [49, 50]. The collapse of the primary and secondary health facilities has put serious pressure on tertiary health facilities that are not optimally prepared [51]. Most patients were initially seen in primary care, and diagnosis was confirmed for all patients at the tertiary care level [36, 37, 39, 42, 52, 53]. Referral to secondary or tertiary care for diagnostic confirmation is frequently delayed, however, because primary care physicians in LMICs misdiagnose lung cancer and misinterpret chest CT scan and thereafter administer ATT which creates a false sense of security in the patients and their families, thereby delaying referral [34, 35, 39, 42]. As the number of doctors visited increased, referral delay, doctor delay, and total delay also increased [37, 40]. The roles of multidisciplinary team approach was underemphasized in the studies, basic support staff who are needed for cancer care like the oncology nurses, laboratory scientists, pulmonary specialists, thoracic surgeon, those trained in palliative care, and other health-care workers are not available to provide modern oncology services and these results in delays in timely lung cancer care continuum from diagnosis to palliative care [21, 54, 55]. Increased focus on health system delay is necessary if we want to end the delays in timely lung cancer diagnosis to palliative care in LMICs, including SSA.

### Strengths and limitations of this study

The advantage of conducting a scoping review was evident in how it highlighted the dearth of evidence on the health systems issues impacting on the delays in timely lung cancer care continuum from diagnosis to palliative care in LMICs, thereby identifying potential research gaps and future research needs. The systematic nature of the searches using different database and different searching strategies (manual and electronic) were the most important strength of this study. An important limitation is that, although a thorough search of the literature was conducted using clear inclusion and exclusion criteria, it is possible that relevant articles were missed particularly given that reviewers involved in study selection searched independently. The review was limited to studies published in English, as it is the commonly used language for communication in most LMICs. Our included studies were limited to articles published from January 2008 to June 2018 because the literature searches showed that most relevant studies were conducted after 2008. The review focused on articles published in LMICs, due to comparable settings and similar resources available.

## Conclusions

This scoping review of literature highlighted the health systems influence on timely diagnosis of lung cancer continuum from diagnosis to palliative care. The health systems pathways of care for lung cancer should begin with prevention and continue through all stages of diagnosis to palliative care. A functioning health care system is fundamental to the achievement of universal coverage for health care. Reducing patient wait time across this care continuum is needed to improve easy access to healthcare, quality care, survival and patient outcomes, as many patients still face longer wait times for diagnosis and treatment of lung cancer than recommended in several healthcare settings. A multidisciplinary team approach will help to reduce wait time and ensure that all patients receive appropriate care. Interventions are needed to address delays in lung cancer care in LMICs. There is need for financial investments and more research on lung cancer care continuum in LMICs, including SSA. Building diagnostic capacity and improving referral mechanisms can overcome common barriers to timely lung cancer diagnosis. Health-care providers at all levels of care should be educated and equipped with skills to identify lung cancer symptoms and perform or refer for appropriate diagnostic tests.

It is important for LMICs, including SSA to strengthen their health-care systems by ensuring that it has an adequate screening infrastructure to enable all appropriate patients get tested, make certain that the GPs are educated about lung cancer, undertake a public education campaign to raise awareness of lung cancer symptoms and the importance of early diagnosis to reduce delays to timely diagnosis.

## Supplementary information

**Additional file 1.** Search strategy.

**Additional file 2.** Degree of agreement calculation.

**Additional file 3.** Table 1: Quality assessment of included studies.

## Data Availability

All data generated or analysed during this study are included in this published article and its supplementary information files.

## Abbreviations

ATT: Antitubercular treatment
BTS: British Thoracic Society
GP: General practitioner
HSSREC: Humanities & Social Sciences Ethics Committee
LMICs: Low- and Middle-Income Countries
MeSH: Medical Subject Headings
MMAT: Mixed Method Appraisal Tool
NSCLC: Non-Small Cell Lung Cancer
PRISMA: Preferred Report Items for Systematic and Meta-Analysis
SDGs: Sustainable Development Goals
SSA: Sub-Saharan Africa
UHC: Universal Health Coverage
UKZN: University of KwaZulu-Natal
WHO: World Health Organization
