# Supplementary material for: Health systems influence on the pathways of care for lung cancer in low- and middle-income countries: a scoping review
Source: Global Health. 2020 Mar 18;16:23. doi: 10.1186/s12992-020-00553-8 (PMC7081618; doi:10.1186/s12992-020-00553-8)
Supplement: Supplementary file 1 — Additional file 1. Search strategy. [file 12992_2020_553_MOESM1_ESM.docx]

**Additional file 1**

**Appendix 1.** Search strategy.

(("lung neoplasms"[MeSH Terms] OR ("lung"[All Fields] AND "neoplasms"[All Fields]) OR "lung neoplasms"[All Fields] OR ("lung"[All Fields] AND "cancer"[All Fields]) OR "lung cancer"[All Fields]) AND ("diagnosis"[Subheading] OR "diagnosis"[All Fields] OR "diagnosis"[MeSH Terms])) AND ("delivery of health care"[MeSH Terms] OR ("delivery"[All Fields] AND "health"[All Fields] AND "care"[All Fields]) OR "delivery of health care"[All Fields] OR ("health"[All Fields] AND "care"[All Fields] AND "system"[All Fields]) OR "health care system"[All Fields]) AND ("2008/01/01"[PDat] : "2018/06/11"[PDat] AND "humans"[MeSH Terms]).
