# Supplementary material for: Health systems influence on the pathways of care for lung cancer in low- and middle-income countries: a scoping review
Source: Global Health. 2020 Mar 18;16:23. doi: 10.1186/s12992-020-00553-8 (PMC7081618; doi:10.1186/s12992-020-00553-8)
Supplement: Supplementary file 2 — Additional file 2. Degree of agreement calculation. [file 12992_2020_553_MOESM2_ESM.docx]

**HEALTH SYSTEMS INFLUENCE ON THE PATHWAYS OF CARE FOR LUNG CANCER IN LOW-AND MIDDLE-INCOME COUNTRIES: a scoping review.**

Full Article Screening

|  | Author and Year | Reviewer 1: Response | Reviewer 2: Response |
| --- | --- | --- | --- |
| 1 | Abrao Fernando Conrado et al and 2017 | 1 | 1 |
| 2 | Chandra Subhash et al and 2009 | 1 | 1 |
| 3 | Chatterjee, Surajit et al and 2016 | 1 | 1 |
| 4 | Fernandez de la Vega, J. F. et al and 2015 | 1 | 0 |
| 5 | Hsieh Vivian Chia-Rong et al and 2012 | 1 | 1 |
| 6 | Ramachandran Krishnappriya et al and 2016 | 0 | 1 |
| 7 | Sulu Ebru et al and 2011 | 1 | 1 |
| 8 | Valdés Solangel et al and 2010 | 1 | 1 |
| 9 | Yurdakul Ahmet Selim et al and 2015 | 1 | 1 |
| 10 | Živković Danko and 2014 | 1 | 1 |

CALCULATIONS FOR DEGREE OF AGREEMENT USING STATA 13

**Stata output based on the above results**

Expected

Agreement Agreement Kappa Std. Err. Z Prob>Z

-----------------------------------------------------------------

80.00% 82.00% -0.1111 0.3162 -0.35 0.6373

| Controls |

Cases | Exposed Unexposed | Total

-----------------+------------------------+------------

Exposed | 8 1 | 9

Unexposed | 1 0 | 1

-----------------+------------------------+------------

Total | 9 1 | 10

McNemar's chi2(1) = 0.00 Prob > chi2 = 1.0000

Exact McNemar significance probability = 1.0000

Proportion with factor

Cases .9

Controls .9 [95% Conf. Interval]

--------- --------------------

difference 0 -.3771808 .3771808

ratio 1 .734931 1.360672

rel. diff. 0 -2.771808 2.771808

odds ratio 1 .0127394 78.49684 (exact)

**Results interpretation**

Analysis of the results of full article screening show that there was 80.00% agreement versus 82.00% expected by chance which constitutes a considerably poor agreement between screeners (Kappa statistic = - 0. 11 and p-value <0.05). However, the McNemar's chi-square statistic suggests that there is not a statistically significant difference in the proportions of yes/no answers by reviewer with p-value <0.05.
